# The Spatiotemporal Dynamics of Conditioned Behavior: First-Order and Higher-Order Conditioning

**DOI:** 10.1037/xan0000392

**Published:** 2025-04

**Authors:** Victor M. Navarro, Dominic M. Dwyer, Robert C. Honey

**Affiliations:** 1 School of Psychology, University of Cardiff

**Keywords:** rat, goal-tracking, sign-tracking, timing

## Abstract

Pavlovian conditioning procedures generate spatially and temporally distinct behaviors. For example, after rats have received pairings of a lever with food, they approach the food well during the lever (called goal-tracking) and interact with it (called sign-tracking), with these two spatially distinct behaviors being distributed differently across the temporal duration of the lever. Experiment 1 assessed the development of these spatiotemporally defined behaviors during first-order conditioning, as a function of the sequence in which the lever and food occurred (lever→food or food→lever) and the interval between them (1 s or 11 s). In Experiment 2, the same rats received higher-order conditioning trials in which an auditory stimulus was paired with the lever and the emergence of goal-tracking to the auditory stimulus was assessed. The results of Experiments 1 and 2 revealed dissociations between where and when learning was evident during first- and higher-order conditioning, underscoring the need for models of Pavlovian conditioning to explain both the nature and timing of different conditioned responses.

Most descriptions of Pavlov’s conditioning studies with dogs emphasize the fact that a conditioned stimulus (CS; e.g., a ticking metronome) comes to elicit a similar response to the unconditioned stimulus (US; e.g., the delivery of food into a bowl) as a consequence of their pairing. In these studies, dogs were placed in a harness constraining their movements to enable the accurate collection of drops of saliva from the dogs’ mouths during the CS (the conditioned response or CR). However, such descriptions of the consequences of conditioning are a caricature of Pavlov’s original observations. For example, the presentation of conditioned stimuli also prompted orienting to the food bowl, and when the dogs were unharnessed, they approached the location in which the CS was located as it was moved from one position in the experimental room to another (e.g., Pavlov, 1928; p. 168; see also Zener, 1937). Moreover, the salivary CR, which was assessed across the temporal duration of the CS, was less marked at the start of the CS than at its end (Pavlov, 1928, p. 149); a phenomenon known as inhibition of delay.

The fact that Pavlovian conditioning procedures generate spatiotemporally distinct behaviors, including those related to the nature of the US (e.g., the delivery of food into a bowl) and the CS (e.g., its location in the experimental room), has been confirmed in many preparations (e.g., Holland, 1977, 1984; Iliescu et al., 2020; Timberlake & Grant, 1975). Such findings mean that key questions about the conditions, content, and mechanisms of Pavlovian conditioning (see Dickinson, 1980; Rescorla, 1988) cannot be answered adequately by measuring a single conditioned behavior: The answers based on one measure might not generalize to other behaviors either within a given preparation (i.e., be internally coherent; see Patitucci et al., 2016) or across preparations (i.e., be externally coherent). Yet this is precisely the approach that has dominated assessment of predictions generated by theoretical models of conditioning processes (e.g., Mackintosh, 1975; Pearce, 1994; Pearce & Hall, 1980; Rescorla & Wagner, 1972; Stout & Miller, 2007; Wagner, 1981), from conditioned suppression in rats (e.g., Dickinson et al., 1976; Hall & Pearce, 1979; Kasprow et al., 1987; Rescorla, 1968) to autoshaped key pecks in pigeons (e.g., Leyland & Mackintosh, 1978; Swan & Pearce, 1987). To be clear, this methodological approach to testing predictions derived from theoretical models has an important limitation: Because the models have little to say about how their underlying constructs (e.g., the strength of a CS→US association, V_CS_) affect the spatiotemporal properties of conditioned behaviors, it is unclear whether their predictions are internally coherent. Do their predictions hold across all measures of conditioning in each preparation or only a subset, and if only a subset, then why that subset? Recent research reinforces this critique.

Navarro et al. (2023) reported the results of a series of experiments using an autoshaping procedure with rats in which the temporary introduction of a lever into the experimental apparatus served as the CS and the delivery of a food pellet into a food well was the US. The procedure is an example of Pavlovian conditioning, with the US being delivered independently of the behavior of the rat. In one experiment, two groups of rats received presentations of two levers, L1 and L2, which were separately introduced into the experimental chamber for 10 s and then withdrawn. In group forward, L1 was followed by the delivery of a food pellet into a food well, whereas L2 was not paired with food. In group intermixed, L1 was followed by food on half of its presentations and preceded by food on the remainder, and L2 was not paired with food. Discrimination learning was assessed using two behavioral measures: visiting the food well during L1 and L2 (called goal-tracking; e.g., Boakes, 1977; Good & Honey, 1991) and interacting with L1 and L2 (called sign-tracking; e.g., Hearst & Jenkins, 1974; see also Davey & Cleland, 1982). These two responses are depicted for the presentation of a single lever in Figure 1. L1 came to elicit more goal-tracking than L2, and this difference emerged every bit as readily in groups forward and intermixed. L1 also came to elicit more sign-tracking than L2, but this difference was more evident in group forward than in group intermixed. This example illustrates the fact that any answer to key questions about the conditions, content, and mechanisms of learning will differ depending on the measure of learning that is used: The impact of the two training procedures, forward conditioning versus intermixed forward and backward conditioning, depends on which behavioral index is used (goal-tracking or sign-tracking). This indeterminacy is reinforced by the fact that the temporal profiles of these two conditioned behaviors across the temporal duration of the levers were quite different. In group forward, goal-tracking increased across the duration of a 10-s lever, whereas sign-tracking declined; in group intermixed, the levels of goal-tracking were consistently high and those of sign-tracking were consistently low across L1 (Navarro et al., 2023; see also Iliescu et al., 2020). Why should learning be more evident in one behavioral measure (e.g., goal-tracking) at one point during the CS and in a different measure (e.g., sign-tracking) at another point?[Fig fig1]

The questions raised by group-level dissociations in how learning is expressed proliferate once it is recognized that there are also individual differences in how learning is expressed. Pavlov (1928) observed marked quantitative and qualitative individual differences in the effects of conditioning in dogs. More recently, Patitucci et al. (2016; see also Flagel et al., 2009) showed that while some rats given forward conditioning trials with L1 and nonreinforced presentations of L2 exhibited learning primarily as differences in goal-tracking during L1 and L2, others exhibited it as differences in sign-tracking. Given the fact that all rats received the same experimental training, why should there be both quantitative and qualitative differences in how learning is expressed? These observations are quite beyond general-process models that make the simplifying assumption that there is an ordinal mapping between theoretical constructs (e.g., associative strength, V) and conditioned behavior (e.g., Rescorla & Wagner, 1972; see also Mackintosh, 1975; Pearce & Hall, 1980).

The issues raised above have started to be addressed by an associative model of Pavlovian conditioning, how excitation and inhibition determine ideo-motion (HeiDI; Honey & Dwyer, 2022; Honey et al., 2020; Navarro et al., 2023). Indeed, the results presented by Navarro et al. (2023), involving the efficacy of backward conditioning procedures, provide key support for HeiDI. HeiDI assumes that forward conditioning trials (e.g., lever→food) result in the formation of reciprocal associations between the lever and food (i.e., a lever→food association and a food→lever association, or lever⇄food associations). This assumption means that the strength of both reciprocal associations can contribute to the generation of conditioned behaviors (see also Asratyan, 1965), and it predicts that backward conditioning trials (e.g., food→lever) will engender conditioned responding when the lever is tested alone, based on food⇄lever associations. Experiment 1 sought to replicate and extend the results reported by Navarro et al. (2023) using a between-subjects design, by examining the impact of (a) forward (lever→food) and backward (food→lever) conditioning trials, and (b) the interval between the lever and food (1 s or 11 s), on the distribution of goal-tracking and sign-tracking across the duration of the lever. In Experiment 2, the same rats received higher-order conditioning trials in which an auditory stimulus (S1) was paired with the conditioned lever, and we examined the development of goal-tracking to S1 and its temporal distribution across S1. HeiDI provides a basis for the paradoxical prediction that arranging a trace interval between the lever and food, on forward conditioning trials, will increase higher-order conditioning to S1 (cf., Lin et al., 2013; Lin & Honey, 2011). The derivation of this prediction (formally described in Honey & Dwyer, 2022) will be presented in the context of Experiment 2, alongside alternative theoretical analyses.

## Experiment 1

The design of Experiment 1 is depicted in Table 1. Rats received trials on which presentations of L1 (e.g., the lever to the left of the food well) were paired with food (L1→food) and those of L2 (e.g., the lever to the right of the food well) were not (L2→no food). We manipulated whether rats received forward conditioning (L1→food) or backward conditioning (food→L1) trials with L1, and whether the interval between the offset of L1 and the presentation of food (on forward trials), or the presentation of food and the onset of L1 (on backward trials) was 1 s or 11 s. This factorial design resulted in four groups: forward-1s, forward-11s, backward-1s, and backward-11s. All groups received nonreinforced presentations of L1 in each session, providing an assessment of conditioned responding to the lever in the absence of responding generated by the presentation of food (which is a concern in the backward-1s and backward-11s groups). Trace conditioning typically impairs the development of a given conditioned behavior, with the impact of the trace interval depending on the conditioning preparation (for a review, see Mackintosh, 1974). In contrast, the development of excitatory Pavlovian conditioning generated by backward conditioning has often been cast as either evanescent (e.g., Heth, 1976) or unreliable (see Mackintosh, 1974, p. 60). However, some evidence suggests that the efficacy of backward conditioning trials can be revealed using procedures that allow animals to express the fact that backward associations (like forward associations) encode information about the temporal order of the US and CS (e.g., Arcediano et al., 2005; see also Asratyan, 1965, pp. 178–179). Moreover, Navarro et al. (2023) showed that even simple backward conditioning trials (in which the presentation of food preceded a lever by 1 s) can produce sustained and marked evidence of excitatory conditioning as indexed via goal-tracking but not sign-tracking, while also noting that longer US–CS intervals produce inhibitory conditioning (Delamater et al., 2003; Taira et al., 2024). Notably, Navarro et al.’s procedure used a relatively short intertrial interval (ITI) (60s on average), which might have favored the expression of goal-tracking (Thomas & Papini, 2020). Here, we used a longer ITI (130s on average) with the hope of generating more equivalent levels of the two forms of conditioned responding. This change should allow the baseline levels of goal-tracking (conditioned or not) to decline or extinguish (see also Cinotti et al., 2019).[Table tbl1]

### Method

#### Subjects

Sixty-four naïve male Lister Hooded rats (mean ad lib weight = 342 g; range: 309–373 g; supplied by Envigo, United Kingdom) were randomly assigned to each of the four groups (*n* = 16 per group). No formal power analysis was conducted because it was not possible to estimate the effect sizes for both first-order and higher-order conditioning based on immediately relevant prior research. Instead, the number of subjects was based on prior research that we have conducted using the autoshaping procedure (e.g., Navarro et al., 2023; Patitucci et al., 2016). Rats were housed in pairs in standard home cages. They were maintained between 85% and 95% of their ad lib weights by giving them restricted access to food at the end of each day in these cages, where they had continuous access to water. The room in which these home cages were placed had a 12-hr/12-hr light/dark cycle (lights on at 7 a.m.). The research was conducted in accordance with Home Office regulations under the Animal (Scientific Procedures) Act 1986. Research was conducted in accordance with the Home Office regulations under the Animal (Scientific Procedures) Act 1986 and under the authority of project licence number PP3468526 granted to D. M. Dwyer.

#### Apparatus

Sixteen identical conditioning boxes (30 × 24 × 21 cm: H × W × D; Med Associates, Georgia, Vermont) were used, with each box being placed in a sound-attenuating shell incorporating a ventilation fan that maintained the background noise at 68 dB(A). The boxes had two aluminum side walls, with front walls, back walls, and ceilings made from clear acrylic. The floor of each box was formed from 19 steel rods (4.8 mm diameter, 16 mm apart) placed above a stainless-steel tray. Food pellets (45 mg; LabDiet, St. Louis, Missouri, United States) were delivered to a food well (aperture: 5.3 × 5.3 cm), which was recessed in the center of the left wall at floor level. The food well was equipped with infrared detectors. Both the onset and offset of interruption (e.g., by a rat’s snout in the food well) of the detector were registered, allowing for duration of food well responses to be recorded. Two retractable levers (4.5 × 1.8 × 0.2 cm), located 3 cm to the left and right of the food well, were positioned at a height of 4.6 cm and 1.5 cm from the edge of the walls. As with the food well, we recorded a signal when the levers were depressed by 4 mm from their horizontal resting position and again when they were released back to their resting position, which allowed the computation of the duration of each lever press. Each box was equipped with a speaker mounted behind the wall facing the levers/food well and 18 cm above the floor. In Experiment 2, these speakers delivered auditory stimuli (white noise and a 5-Hz clicker) at an intensity of 6 dB above background sound levels. Med-PC^®^ (Med Associates Inc.) software controlled the insertion and retraction of the levers, delivery of food pellets and auditory stimuli, and recorded food well entries and lever presses. Presentations of the levers and auditory stimuli were synchronized to occur simultaneously across all boxes.

#### Procedure

Rats received one 46-min pretraining session in which 20 food pellets were delivered on a variable time (VT) 130-s schedule (range: 110–150 s). On each of the following 20 days, rats received a single training session that occurred at the same time of day for a given rat (with the earliest session starting at 09:00 a.m.). The first 10 trials in each session included five 10-s presentations of L1 and L2, with the assignment of the left and right levers to L1 and L2 counterbalanced. For rats in groups forward-1s and forward-11s, a single food pellet was delivered 1 s and 11 s, respectively, after L1 was retracted from the chamber. For rats in group backward-1s and backward-11s, a single food pellet was delivered 1 s and 11 s, respectively, before L1 was extended into the chamber. For all rats, presentations of L2 were not paired with food. The final 12 trials in each session included eight trials as described above (four with L1 and four with L2), and four nonreinforced probe trials (two with L1 and two with L2) in which no pellets were delivered (i.e., L1 like L2 was neither preceded nor followed by food). The trials were delivered on a VT 130-s schedule (range: 110–150s). The order in which the two levers were presented was random with the constraint that there were no more than two presentations of the same lever in succession.

#### Data Analysis

All analyses were performed in R (R Development Core Team, 2021), using packages brms (Bürkner et al., 2022), and bayestestR (Makowski et al., 2022). To facilitate reproducibility, a singularity image with the R environment used for the analyses is available on the Open Science Framework (OSF) link in the Transparency and Openness section. Files containing raw data were first processed in R to calculate the proportions of goal- and sign-tracking. We defined these proportions as the cumulative time in which a response was recorded (the total duration of all nose pokes or lever presses over a time window) divided by the length of time the response was measured (10 s for trial-level analyses and 2-s bins for stimulus-level analyses; see ahead). Proportions were used rather than response rates because they are less variable, especially for sign-tracking. Background levels of goal-tracking during the ITI and in the trace intervals between L1 and food were not assessed: Our primary interest was whether L1 and L2 differed (in the context of first-order conditioning in Experiment 1) and whether S1 and S2 differed (in the context of higher-order conditioning in Experiment 2). Comparison of the proportions of responding during the stimuli (L1, L2, S1, and S2) and in their absence is complicated by differential stimulus support for the CRs (i.e., goal-tracking and sign-tracking). For example, while the recent presentation of food would encourage goal-tracking in an immediately succeeding interval, the removal of L1 (or L2) would reduce any competition between sign-tracking and goal-tracking and increase goal-tracking. However, these levels of background and trace goal-tracking are available in the data sets available through the OSF link provided below. Response proportions were regressed using Gaussian processes (GP), whose parameters were estimated under a Bayesian framework. Briefly, GPs model a joint distribution of data points and Gaussian functions, such that each data point is modeled as a linear combination of (indefinite, but data-supported) functions. The estimation of GPs is computationally expensive but has several advantages; crucially for the present work, GPs are a principled way to deal with nonmonotonicities in the data, without specifying an explicit model (c.f., Navarro et al., 2023; see Schulz et al., 2018, for an accessible introduction to GPs). In the present approach, we used GPs to jointly model goal- and sign-tracking proportions using a Dirichlet distribution adopting goal-tracking, sign-tracking, and alternative behaviors as mutually exclusive states, but whenever goal-tracking was the only response measured, or when the two types of response were differentially available across the trial (see Experiment 2), we modeled those probabilities against alternative behaviors using a beta distribution. Each model was estimated via eight chains of 8,000 iterations each (2,000 warmup iterations) and then subjected to convergence and posterior predictive checks (see annotated R code in the OSF repository). After the models were estimated, we performed inference using probability of direction (pd) tests on expected mean posterior differences (MPD). In this context, the pd describes the maximum proportion of posterior differences smaller or larger than zero (no difference). Notably, the pd test is inversely related to *p*-values, such that larger probabilities map onto smaller *p*-values (Makowski et al., 2019), with pds = {.950, .975, .995, .9995} roughly mapping to *p*s = {.1, .05, .01, and .001}, respectively, though pds denote the probability of an effect rather than the probability of observing the measured effect under a null distribution. MPDs provide nonstandardized estimations of effect size, and we quantify the uncertainty surrounding these effect sizes via 95% credible intervals (CIs) on each MPD, which are the Bayesian analogue of frequentist confidence intervals.

#### Transparency and Openness

All data, scripts, and models (and their posterior samples) used for the data analyses reported in this article are available at: https://osf.io/ntkg3/.

### Results

Figure 2 depicts the development of goal-tracking (left-hand panels, A–D) and sign-tracking (right-hand panels, E–H) across training on the nonreinforced probe trials with L1 and L2. Note that for Figure 2 and all remaining figures, individual points correspond to group means, whereas lines and shaded areas correspond to posterior means and CIs, respectively. Consider first the left-hand panels. Early in training, the overall levels of goal-tracking were higher in rats given forward training trials (groups forward-1s and forward-11s, A and B) than in those given backward training trials (groups backward-1s and backward-11s, C and D), MPD = .03, 95% CI [.01, .05], pd = .998 in Block 1. However, this difference was reversed by the end of training, with overall levels of goal-tracking being higher in backward groups than in forward groups, MPD = −.04, 95% CI [−.06, −.02], pd > .999 in Block 5. It is also apparent that while the overall levels of goal-tracking were higher during L1 than L2 in the forward groups (MPD = .02, 95% CI [.01, .04], pd = .998), this was not the case in the backward groups, which showed no reliable differences in goal-tracking during L1 and L2 (MPD = .001, 95% CI [−.01, .01], pd = .539). Now consider the right-hand panels. Sign-tracking levels were higher to L1 than to L2 in both forward groups (E and F; MPD = .06, 95% CI [.04, .08], pd > .999 for group forward-1s and MPD = .02, 95% CI [.01, .04], pd = .997 for group forward-11s) and the backward-1s group (G, MPD = .02, 95% CI [.01, .04], pd > .999). However, there were no reliable differences in sign-tracking to L1 and L2 in the backward-11s group (H, MPD = .01, 95% CI [−.004, .02], pd = .859). Notably, reliable differences in goal-tracking during L1 and L2 were only evident in group forward-11s during Blocks 1 and 2 (smallest MPD = .05, 95% CI [.01, .10], pd = .992 in Block 2). Conversely, reliable differences in sign-tracking to L1 and L2 were detected more frequently, especially later in training. Group forward-1s showed reliable differences in Blocks 2–5 (smallest MPD = .04, 95% CI [.01, .07], pd = .992 in Block 2), group forward-11s did so in Blocks 4 and 5 (smallest MPD = .05, 95% CI [.02, .08], pd > .999 in Block 4) and group backward-1s did so in Blocks 2–5 (smallest MPD = .02, 95% CI [.003, .04], pd = .992 in Block 2). There were no reliable differences in sign-tracking between L1 and L2 in group backward-11s.[Fig fig2]

To assess the distribution of responses across the duration of the levers, we divided the data from the final four sessions of first-order conditioning (Block 5 in Figure 2) into successive 2-s bins relative to the onset of the levers. On this block, the levels of goal-tracking and sign-tracking during L1 and L2 appeared to have reached a point of relative stability. Figure 3 shows those distributions during the L1 and L2 trials. In line with performance toward the end of training, neither of the forward groups showed reliably greater goal-tracking to L1 than to L2 in any bin (A, largest MPD = .01, 95% CI [−.02, .05], pd = .764 and MPD = .01, 95% CI [−.01, .05], pd = .806 for groups forward-1s and forward-11s, both at 10 s).^1^ This pattern of goal-tracking was also true for both backward groups (C–D, largest MPD = .02, 95% CI [−.05, .09], pd = .713 for group backward-1s at 2 s and MPD = .02, 95% CI [−.02, .06], pd = .835 for group backward-11s at 10 s). Conversely, sign-tracking showed reliable differences in nearly all groups. Both forward groups showed reliable differences between L1 and L2 from 2 s to 10 s of lever presentation (E–F, smallest MPD = .04, 95% CI [.01, .08], pd = .994 for group forward-1s at 2 s and MPD = .05, 95% CI [.004, .09], pd = .984 for group forward-11s at 10 s). Notably, the backward-1s group showed increased sign-tracking to L1 starting at 4 s (G, smallest MPD = .02, 95% CI [.002, .05], pd = .984 at 4 s), but the backward-11s group showed no reliable differences (H, largest MPD = .003, 95% CI [−.02, .02], pd = .640 at 10 s).[Fig fig3]

### Discussion

Experiment 1 examined first-order conditioning during a discrimination task in which one lever (L1) was paired with food and a second lever (L2) was not. Overall, increasing the ITI (relative to that used routinely in our previous research) seemed to increase the levels of sign-tracking relative to those that we observe typically (e.g., Navarro et al., 2023; Patitucci et al., 2016), replicating the findings of Thomas and Papini (2020). For rats given forward conditioning trials (i.e., L1→food), differential responding to L1 and L2 was rarely evident in goal-tracking but strongly evident in sign-tracking. Moreover, by the end of training the difference in sign-tracking between L1 and L2 was more evident in group forward-1s than in group forward-11s. That is, the introduction of an 11s trace interval (in group forward-11s) impaired the development of differential responding to L1 and L2 (cf., Honey & Hall, 1992; Pavlov, 1928). For rats given backward conditioning trials (food→L1), differential responding to L1 and L2 was restricted to group backward-1s and the sign-tracking measure. While this effect was not large, it was sustained across many sessions of training. In Experiment 2, we examined the ability of L1 and L2 to support higher-order conditioning as indexed by goal-tracking to two auditory stimuli, S1 and S2.

## Experiment 2

Experiment 2 assessed the extent to which L1 (and L2) supported the development of higher-order conditioning to auditory stimuli S1 (and S2): S1 (e.g., 10 s of white noise) was paired with L1 and S2 (e.g., a 10-s clicker) was paired with L2 (see Table 1). Goal-tracking during S1 and S2 was used to assess higher-order conditioning because it can be measured automatically in the same way as goal-tracking to L1 (and L2); we did not attempt to measure sign-tracking to S1 and S2. While first-order conditioning to a CS is often impaired when there is a long trace interval between that CS and food (see Mackintosh, 1974), a CS established using a trace conditioning procedure can support greater higher-order conditioning than a CS established without such a trace interval (Lin & Honey, 2011; see also Lin et al., 2013). This intriguing observation is beyond standard accounts of higher-order conditioning. It has, however, been addressed in a recent formal model of higher-order conditioning based on HeiDI (Honey & Dwyer, 2022; Navarro et al., 2023).

First, assume that higher-order conditioning reflects the capacity of S1 to evoke the memory of L1, which was itself linked to food during first-order conditioning. Now assume that the L1-food association encodes the intensity of L1 at the point when food was delivered. This intensity will be lower when there is a trace between L1 and food than when there is no trace interval. As a result of initial pairings of S1 with L1, S1 will retrieve a memory of L1 with a low intensity. This low-intensity L1 representation will be more like the intensity of the L1 conditioned with a trace interval than to the intensity of the L1 conditioned with no such interval and should more readily activate the memory of food. On this basis, trace conditioning with L1 might be expected to generate more higher-order conditioning than conditioning with no trace interval (or a shorter interval). Honey and Dwyer (2022) used formal simulations, including a requisite similarity function, to demonstrate the range of conditions under which this prediction holds, which is constrained by the strength of the association between S1 and L1. The comparison of groups forward-1s and forward-11s allows the generality of the effect demonstrated by Lin and Honey (2011) to be investigated in an autoshaping procedure, which was an important impetus for the development of HeiDI. It is worth noting that the predicted results for Experiment 2 (and those reported by Lin & Honey, 2011) are inconsistent with the temporal coding hypothesis. According to this hypothesis, associations between stimuli encode their temporal order, and different temporally coded associations (e.g., L1→1s→food and S1→L1) can be integrated through a common referent, in this case, the referent would be L1. The integration of the temporally coded associations in group forward-1s, L1→1s→food and S1→L1, should result in S1 and food being relatively coincident when S1 is presented (i.e., S1→11s→food; 11 s because L1 is 10 s and there is a 1-s gap between the offset of L1 and food). In contrast, in group forward-11s, the integration of S1→L1 and L1→11s→food should result S1 and food being less coincident (i.e., S1→21s→food). The temporal coding hypothesis predicts that trace conditioning should disrupt higher-order conditioning.

The temporal coding hypothesis provides a clear prediction about our backward conditioning procedures. In group backward-1s, the food→1s→L1 association can be integrated with a S1→L1 association through the referent, L1, so that when S1 is presented it aligns with food. Under these conditions, the hypothesis predicts that S1 would generate conditioned behavior. In contrast, for group backward-11s integrating the food→11s→L1 association with a S1→L1 association would result in S1 being aligned with the trace period and would not be predicted to generate conditioned behavior. There is, however, an alternative analysis for the pattern of results predicted by the temporal coding hypothesis: S1 could evoke a memory of L1 as the result of the S1→L1 association, and to the extent that backward conditioning results in the formation of reciprocal food⇄L1 associations then S1 should generate more conditioned behavior in group backward-1s than backward-11s (Honey & Dwyer, 2022; Honey et al., 2020; Navarro et al., 2023). Finally, to the extent that backward conditioning with a 11-s trace interval in group backward-11s results in the development of inhibition to L1 (Delamater et al., 2003; Taira et al., 2024), then one might expect S1 to gain higher-order conditioned inhibition as a result of being paired with L1 (see Rescorla, 1976), with the influences of such inhibition being evident as low levels of conditioned responding during S1.

### Method

#### Subjects, Apparatus, and Procedure

The rats and apparatus were those described in Experiment 1. On the 12 days that followed Experiment 1, rats continued to receive conditioning trials with L1 and L2 during the first 10 trials (five per lever, as described in Experiment 1), but higher-order conditioning trials were intermixed during the last 12 trials. On higher-order conditioning trials, one of the auditory stimuli, S1 (white noise or clicker, counterbalanced), preceded the presentation of L1, and the remaining auditory stimulus, S2, preceded L2; no food pellets was delivered during these trials. The last 12 trials within each session were subdivided into three, four-trial blocks, each containing one conditioning trial with L1 (varying across groups in the same way as during first-order conditioning), a nonreinforced presentation of L2, and nonreinforced trials on which the offset of S1 was followed by L1 and the offset of S2 was followed by L2 (i.e., S1→L1 and S2→L2 trials). All other experimental parameters were identical to those described for first-order conditioning in Experiment 1.

### Results

Figure 4 shows the proportions of goal-tracking to S1 and S2 across two-session blocks of higher-order conditioning, when S1 preceded L1 and S2 preceded L2 (i.e., S1→L1 and S2→L2). Higher-order conditioning had markedly different effects in the forward groups. For group forward-1s, goal-tracking during S1 was not reliably greater than during S2 on any block (A, highest pd = .937 in Block 5; but pooling data over more sessions did reveal some significant differences, see ahead). In contrast, for group forward-11s, goal-tracking to S1 was reliably higher than to S2 from Block 2 onwards, being numerically largest and statistically most reliable in Block 5 (B, MPD = .04, 95% CI [.01, .08], pd = .997). The two backward groups showed similar levels of goal-tracking during S1 and S2 (C–D, highest pds = .830 in Block 6 and .716 in Block 5 for groups backward-1s and backward-11s, respectively).[Fig fig4]

Figure 5 depicts the distributions of responding during the components of the S1→L1 and S2→L2 trials. The data from each trial were divided into successive 2-s bins relative to the onset of the levers and pooled over the final six sessions of higher-order conditioning (i.e., Blocks 4, 5, and 6 in Figure 4). On these blocks, the levels of goal-tracking and sign-tracking during S1, S2, L1, and L2 appeared to be relatively stable. As the two responses were differentially available across the trial, we assessed these data by estimating two separate models, one for goal-tracking and one for sign-tracking. Goal-tracking in group forward-1s tended to be low during the initial bins of S1 and S2, but increased midway through S1 significantly surpassing goal-tracking to S2 (A–B, largest MPD = .03, 95% CI [.01, .06], pd = .997 at −4s), and later decreased so there was no difference between S1 and S2 (MPD = .02, 95% CI [−.005, .04], pd = .931 at 0 s). Goal-tracking in group forward-11s tended to be similar throughout S1 (and significantly greater than throughout S2; smallest MPD = .03, 95% CI [.01, .06], pd = .992 at 0 s). After the onset of the lever stimuli, goal-tracking to L1 was suppressed, with group forward-1s goal-tracking to L1 being significantly lower than to L2 at 6 s (A, MPD = −.02, 95% CI [−.03, −.0004], pd = .978, likely due to the high levels of sign-tracking in this group during that period) and group forward-11s failing to show any significant differences between L1 and L2 (B, largest MPD = −.01, 95% CI [−.03, .01], pd = .861 at 6 s). Sign-tracking in both forward groups was significantly greater to L1 than to L2 throughout the lever duration (E–F, smallest MPD = .05, 95% CI [.02, .09], pd > .999 for group forward-1s, and MPD = .06, 95% CI [.03, .09], pd > .999 for forward-11s, both at 2 s).[Fig fig5]

The two backward groups showed markedly different patterns of responding during these higher-order conditioning trials. Goal-tracking in group backward-1s was reliably higher to S1 than to S2 at 0 s, during the last 2 s of the auditory stimuli (C, MPD = .04, 95% CI [.01, .07], pd = .998) and reliably higher to L1 than to L2 during 2–4 s (smallest MPD = .04, 95% CI [.01, .07], pd = .993 at 4 s). This is surprising, given the probe trials for L1 and L2 during Experiment 1 revealed no such differences in goal-tracking. In contrast, group backward-11s showed small but reliable differences during the last 2 s of the auditory stimuli and the first 2 s of the levers, with goal-tracking toward the end of S2 being reliably higher than S1 (D, smallest MPD = −.03, 95% CI [−.06, −.001], pd = .979 at 0 s) and goal-tracking during the beginning of L2 being reliably higher than the equivalent to L1 (MPD = −.03, 95% CI [−.05, −.001], pd = .979 at 2 s). Most notably, sign-tracking in both groups given backward conditioning increased and then decreased across L1, becoming reliably higher than sign-tracking to L2 at 8 s of the lever presentation (G–H, MPD = .02, 95% CI [.001, .05], pd = .979 for group backward-1s and MPD = .03, 95% CI [.01, .05], pd = .997 for group backward-11s) but showing no reliable differences at 10 s (MPD = .03, 95% CI [−.002, .06], pd = .963 for group backward-1s and MPD = .02, 95% CI [−.0001, .05], pd = .974 for group backward-11s).

### Discussion

The patterns of higher-order conditioning in groups given either forward or backward first-order conditioning raise several interesting issues, which will be considered in turn. Excitatory higher-order conditioning, as measured by more goal-tracking during S1 than S2, was evident in group forward-11s, but not clearly seen in group forward-1s (Figure 4A and 4B). This observation is surprising given the fact that first-order conditioning with L1 was less evident, as indexed by sign-tracking, in group forward-11s than in group forward-1s (Figure 2E and 2F). However, as already noted, these results have a precedent. Lin and Honey (2011; see also Lin et al., 2013) showed that longer trace intervals generated more higher-order conditioning (second-order conditioning and sensory preconditioning) than shorter intervals. As outlined when introducing Experiment 2, HeiDI provides one potential analysis for this finding (see Honey & Dwyer, 2022). The higher-order conditioning in group forward-11s has an interesting feature: Differences in goal-tracking to S1 and S2 were evident at a point where (differential) first-order conditioning to L1 and L2 was most apparent in sign-tracking. This fact suggests that higher-order conditioning reflected the operation of an associative chain (i.e., S1→L1→food) or a mediated association between S1 and food (i.e., S1→food) rather than a stimulus→response association (i.e., S1→interact with lever; cf., Holland, 1977; Rizley & Rescorla, 1972). These observations are consistent with studies of second-order conditioning in pigeons (e.g., Stanhope, 1992) and sensory preconditioning in rats (e.g., Dwyer et al., 2012), which showed that the nature of higher-order conditioned responding can be quite different from that of first-order conditioning. These results are also consistent with HeiDI, which provides a formal analysis of higher-order conditioning, the conditions under which it occurs and how it is manifest in behavior (Honey & Dwyer, 2022; Honey et al., 2020; Navarro et al., 2023). We will return to HeiDI in the General Discussion section.

Both groups given backward conditioning trials, backward-1s and backward-11s, showed similar levels of goal-tracking during S1 and S2 (Figure 4C and 4D). However, by the later stages of training, group backward-1s showed more goal-tracking during the last seconds of S1 than S2, and more goal-tracking during the initial bins of L1 than L2 (see Figure 5C and 5D). These results provide evidence that backward conditioning, at least in the case of group backward-1s, can produce sustained changes in both sign-tracking (Experiment 1) and goal-tracking (Experiment 2). These results join those reported by Navarro et al. (2023) in showing that backward conditioning can result in sustained changes in excitatory conditioning (cf., Mackintosh, 1974, p. 60; see also Heth, 1976). A final intriguing observation is that in group backward-11s, S1 generated less goal-tracking than S2, and L1 generated less goal-tracking than L2. The lower levels of responding to S1 than S2 are consistent, at least, with the development of inhibitory higher-order conditioning to S1 (Rescorla, 1976), although additional controls would be necessary in order to confirm this interpretation. According to this interpretation, it must be assumed that L1 had gained inhibitory properties, perhaps because it was nonreinforced in a context which had momentarily gained associative strength as the result of the presentation of food (see also Delamater et al., 2003; Taira et al., 2024), and that S1 borrowed these properties through being paired with L1. Of course, according to some models, such borrowing of (inhibitory) associative strength would need to outweigh the fact that the direct link between S1 and food should have been strengthened on S1→L1 trials: Because S1 was nonreinforced in the presence of an inhibitor, L1, the pooled error term within the Rescorla and Wagner (1972) model would be positive, which should have resulted in S1 developing an excitatory association with food. Of course, this is the complement of the argument that is required to explain excitatory second-order conditioning within such a model: The excitatory associative chain (i.e., S1→L1→food) would need to counteract the development of the inhibitory link between S1 and food on nonreinforced S1→L1 trials (for further discussion, see Honey & Dwyer, 2022). The fact that L1 resulted in more sign-tracking than L2 in group backward-11s seems inconsistent with it having inhibitory properties. However, this difference in sign-tracking, which was not evident throughout training in Experiment 1, could be a secondary consequence of the difference in goal-tracking between S1 and S2 rather than evidence of excitatory conditioning. In contrast, for group backward-1s there was consistent evidence of excitatory first-order conditioning from higher levels of both sign-tracking and goal-tracking during L1 than L2 in Experiment 2.

## General Discussion

Pavlovian conditioning is the most widely recognized phenomenon in psychology. However, while some of its properties are well-established, they are often overlooked both descriptively and theoretically. For example, conditioning procedures generate behaviors that not only reflect the nature of the US but also the CS; and these behaviors are temporally distributed across the duration of the CS. Here, we examined the spatiotemporal characteristics of conditioned responding during first-order Pavlovian conditioning (Experiment 1) and higher-order conditioning (Experiment 2) using autoshaping procedures with rats: We measured the development of goal-tracking and sign-tracking across both sessions and the duration of lever presentations, and goal-tracking during the auditory stimuli. One general conclusion that the results of Experiments 1 and 2 reinforce is that the impact of both group-level and within-subjects manipulations critically depends on the nature of the CR that is measured (goal-tracking or sign-tracking) and when it is measured across both sessions and the duration of the stimuli. These dissociations represent a challenge to models of Pavlovian conditioning that assume a monotonic mapping between learning and performance, irrespective of whether they are trial-based (e.g., Mackintosh, 1975; Pearce, 1994; Pearce & Hall, 1980; Rescorla & Wagner, 1972) or time-based (e.g., Gallistel & Gibbon, 2000; Stout & Miller, 2007; Wagner, 1981). Some of these dissociations are highlighted in the next paragraph.

Consider first Experiment 1. Rats received trials on which one lever was paired with food (L1→food), and another lever was not paired with food (L2→no food). Across the later conditioning sessions, differences in responding between L1 and L2 were more evident in sign-tracking than goal-tracking (i.e., forward-1s > forward-11s > backward-1s > backward-11s). The observation that backward and forward conditioning can generate sustained excitatory conditioning is interesting in its own right (cf., Heth, 1976, Mackintosh, 1974, p. 60) and the finding that such conditioning is only evident (here) in sign-tracking does not simply reflect a general lack of sensitivity of goal-tracking as a measure. Most notably, Navarro et al. (2023) used procedures that tended to result in more goal-tracking than sign-tracking to levers (cf., Thomas & Papini, 2020) and observed sustained evidence of both forward and backward conditioning in goal-tracking, but not sign-tracking (see also Iliescu et al., 2018; Patitucci et al., 2016). We turn now to Experiment 2. Rats from Experiment 1 received higher-order conditioning where one auditory stimulus, S1, was paired with L1 (i.e., S1→L1), and S2 was paired with L2 (i.e., S2→L2). Excitatory higher-order conditioning was evident in differential goal-tracking during S1 and S2, with this difference being most clear in group forward-11s (cf., Lin et al., 2013; Lin & Honey, 2011). Evidence consistent with inhibitory higher-order conditioning was evident exclusively in group backward-11s. Moreover, while goal-tracking declined across S1 (and S2) in the backward groups, it did not do so in the forward groups; similarly, goal-tracking across L1 declined in the backward groups and increased in the forward groups (see also Navarro et al., 2023).

How might models of Pavlovian conditioning accommodate such dissociations? We have developed one approach that seems promising. HeiDI assumes that bidirectional associations form during pairings of one a CS (X) and US (i.e., X⇄food), and that pairing a second CS (A) with X allows A to enter into a direct inhibitory association with the US and to access the first-order bidirectional associations (i.e., X⇄food; see Honey & Dwyer, 2022; Honey et al., 2020). To explain differences in the spatial distribution of responding, HeiDI assumes that the relative perceived intensities of the CS and (retrieved) US determines whether CS-oriented responding (e.g., sign-tracking) or US-oriented responding (e.g., goal-tracking) will dominate, with the balance shifting from goal-tracking to sign-tracking as the perceived intensity of the CS increases relative to the US. To explain differences in the temporal distribution of responding, HeiDI assumes that the perceived intensity of the CS declines across its duration and that the perceived intensity at the point when the US arrives becomes associated with that US. One consequence of these assumptions is that associative strength will accrue to a lower perceived intensity during forward than backward conditioning (and contribute to their distinct profiles of responding), with other perceived intensities of the same CS being capable of generating responding to the extent that they are similar (see Navarro et al., 2023). We have provided formal simulations illustrating the general utility of this approach across a broad range of phenomena involving both first-order conditioning and higher-order conditioning—phenomena that were difficult for alternative analyses to address.^2^ These phenomena, which have counterparts in Experiments 1 and 2, included the facts that (a) backward conditioning procedures can support sustained excitatory conditioning, which has a different temporal profile to forward conditioning (Navarro et al., 2023), and (b) excitatory higher-order conditioning can be more effective after first-order trace conditioning (Lin et al., 2013; Lin & Honey, 2013). For example, in group backward-1s there was a marked decline in goal-tracking across the temporal extents of both L1 and L2 (Figure 3C; see also Navarro et al., 2023), which was complemented by a modest but selective increase in sign-tracking across L1 (Figure 3G). Whereas in group forward-1s, goal-tracking remained low throughout L1 and L2 (Figure 3A) and there was a marked increase in sign-tracking across L1 (Figure 3E). Also, first-order forward conditioning (assessed using sign-tracking) was more evident in group forward-1s than group forward-11s (Figure 2E and 2F, respectively), but higher-order conditioning (as assessed using goal-tracking) was more evident in group forward-11s than group forward-1s (Figure 4A and 4B, respectively).

We will close this discussion by considering one feature of the HeiDI model that we have given little formal or indeed informal consideration to in the past: The role of the ITI in learning and performance. In keeping with some others, we have assumed that such intervals provide (further) opportunities for learning about the constellation of features collectively referred to as the experimental context. The perceived intensity of these features might decline (i.e., habituate) across an experimental session, gain and lose associative strength, and generate a range of different behaviors (e.g., exploration, orienting, and sniffing). However, renewed consideration of the role of the ITI is prompted by the observation that in contrast to our previous autoshaping research (with short ITIs), here we observe marked levels of sign-tracking relative to goal-tracking (with longer ITIs; see also Thomas & Papini, 2020). There are several potential explanations for this observation (e.g., within HeiDI). For example, longer ITIs will allow the extinction of any direct (and indirect) associations involving the context (e.g., involving both CSs and USs) and produce changes in the (sign- and goal-tracking) behaviors generated by the context. However, perhaps the most obvious consequence of lengthening the ITI is to reduce the impact of short-term adaptation or habituation on the perceived intensity of the lever and thereby behavior directed toward the lever (i.e., sign-tracking; see Honey et al., 2020). Evaluating this possibility experimentally and presenting formal modeling is beyond the scope of this article (but see Iliescu et al., 2020; Navarro et al., 2023). Nevertheless, being able to shift the distributions of goal-tracking and sign-tracking has the desirable consequence of allowing theoretically important findings (e.g., features of backward conditioning and higher-order conditioning) to be observed with both response measures.

## Figures and Tables

**Table 1 tbl1:** Design of Experiments 1 and 2

Group	Experiment 1		Experiment 2:
	Training trials	Probe trials	Probe trials
Forward-1s	L1→1s→food	L1→no food L2→no food	S1→L1 S2→L2
	L2→no food		
Forward-11s	L1→11s→food		
	L2→no food		
Backward-1s	Food→1s→L1		
	L2→no food		
Backward-11s	Food→11s→L1		
	L2→no food		
*Note.* L1 and L2 denote two levers, and S1 and S2 denote two sounds (a clicker and white noise, counterbalanced). Food indicates the delivery of a food pellet and no food its absence.			

**Figure 1 fig1:**
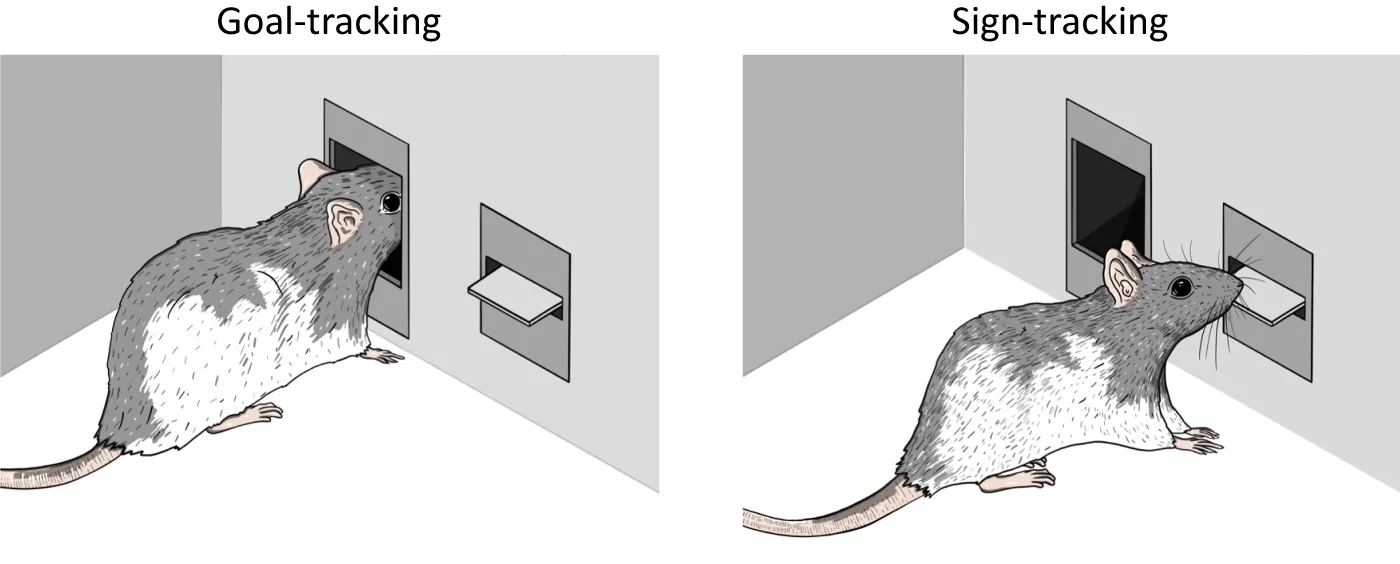
Two Responses Generated by a Pavlovian Conditioning Procedure in Rats in Which the Temporary Insertion of a Lever Into an Experimental Chamber Precedes the Delivery of Food Into a Recessed Food Well *Note.* The rat in the left panel is entering a food well during the lever (called goal-tracking), and the rat in the right panel is interacting with a lever (called sign-tracking). Adapted from “Prediction Error in Models of Adaptive Behavior,” by V. M. Navarro, D. M. Dwyer, and R. C. Honey, 2023, Current Biology, *33*(19), pp. 4238–4243.e3 (https://doi.org/10.1016/j.cub.2023.08.043). CC-BY. See the online article for the color version of this figure.

**Figure 2 fig2:**
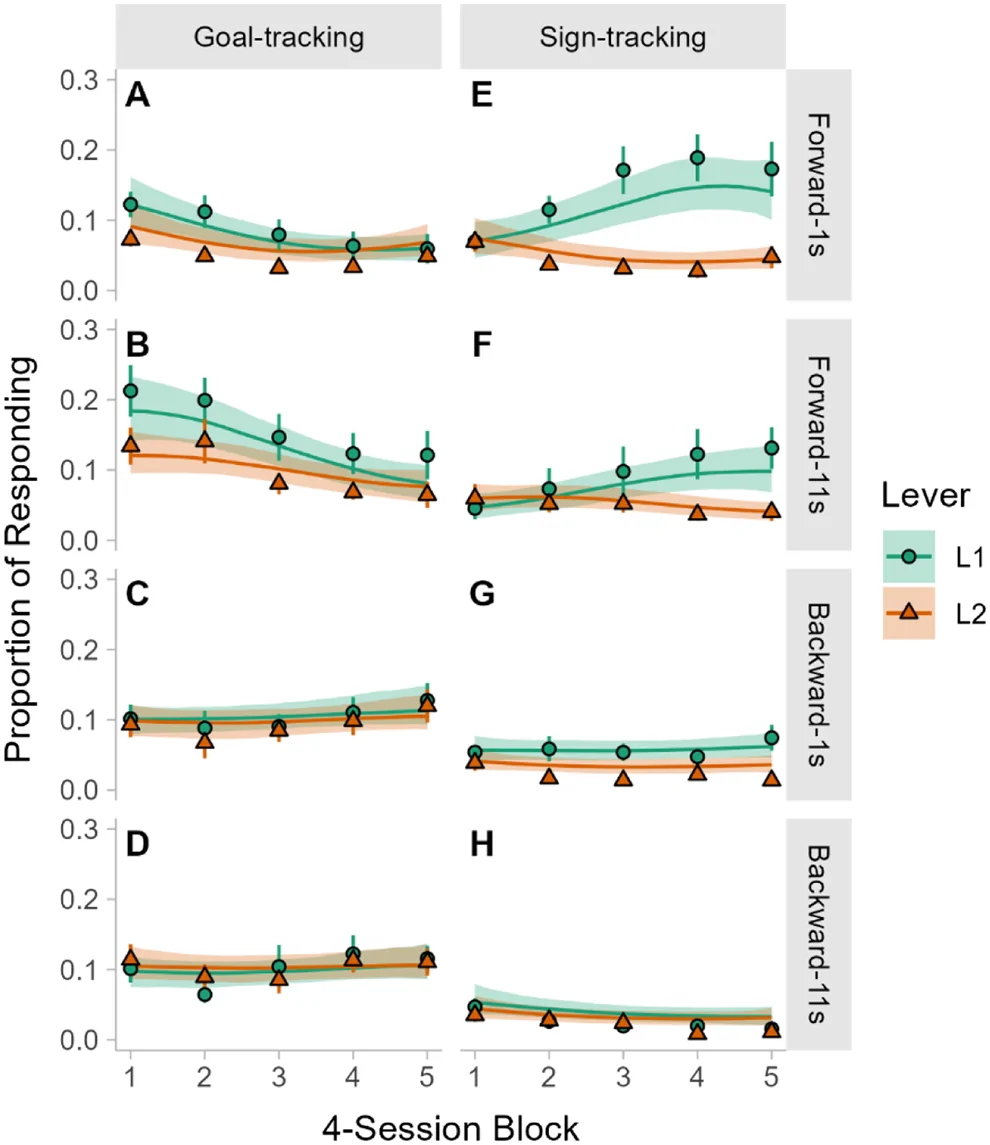
Experiment 1 *Note.* First-order conditioning in four groups of rats distinguished by whether conditioning trials involved forward or backward pairings of L1 and food, and whether the interval between the L1 and food was 1 s or 11 s: forward-1s and forward-11s, backward-1s, and backward-11s. In all groups, L2 was not paired with food. Panels A–D show the proportion of goal-tracking across four-session blocks during nonreinforced probe trials with L1 and L2. Panels E–H show the corresponding proportions of sign-tracking. Results for each group are shown across rows: forward-1s (A and E), forward-11s (B and F), backward-1s (C and H) and backward-11s (D and H). Filled points denote group means and their whiskers ± the standard error of the mean. The lines represent mean posterior predictions from our statistical model, and the shaded areas surrounding them denote their 95% credible interval. L1 = Lever 1; L2 = Lever 2. See the online article for the color version of this figure.

**Figure 3 fig3:**
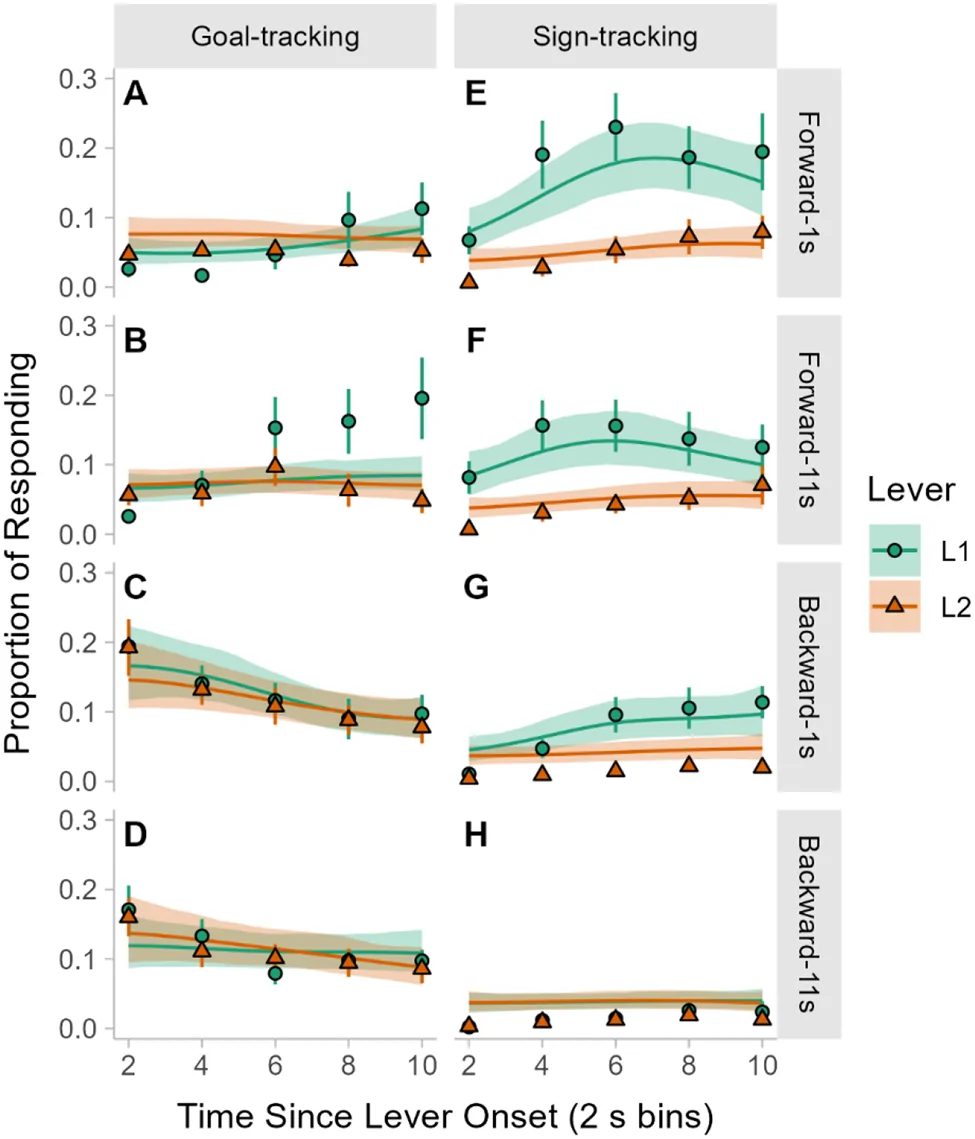
Experiment 1 *Note.* The distribution of responding across 2-s presentations of L1 and L2 during first-order conditioning trials in four groups of rats distinguished by whether conditioning trials involved forward or backward pairings of L1 and food, and whether the interval between the L1 and food was 1 s or 11 s: forward-1s and forward-11s, backward-1s, and backward-11s. In all groups, L2 was not paired with food. Each panel shows the proportion of responding across 2-s bins, during nonreinforced probe trials given on the last four blocks of conditioning. Results for each group are shown across rows: forward-1s (A and E), forward-11s (B and F), backward-1s (C and H), and backward-11s (D and H). Filled points denote group means and their whiskers ± standard error of the mean. The lines represent mean posterior predictions from our statistical model, and the shaded areas surrounding them denote their 95% credible interval. L1 = Lever 1; L2 = Lever 2. See the online article for the color version of this figure.

**Figure 4 fig4:**
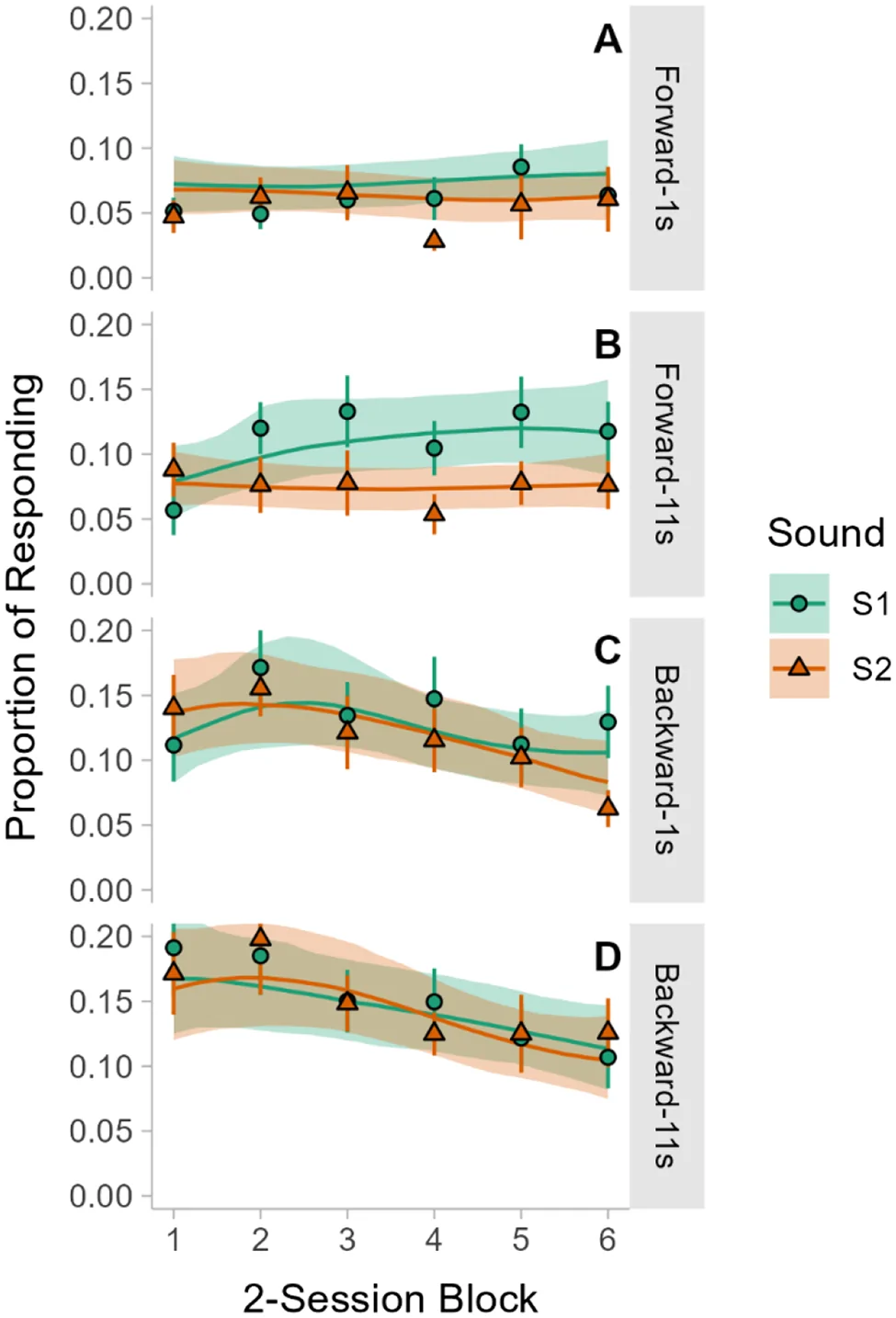
Experiment 2 *Note.* Each panel shows the proportion of goal-tracking during S1 and S2 across two-session blocks of nonreinforced higher-order conditioning trials on which S1 was paired with L1 (S1→L1) and S2 was paired with L2 (S2→L2). The four groups are distinguished by whether first-order conditioning trials involved forward or backward pairings of L1 and food, and whether the interval between the two was 1 s or 11 s: forward-1s and forward-11s (Panels A and B), backward-1s and backward-11s (Panels C and D). Filled points denote group means and their whiskers ± the standard error of the mean. The lines represent mean posterior predictions from our statistical model, and the shaded areas surrounding them denote their 95% credible interval. S1 = Sound 1; S2 = Sound 2; L1 = Lever 1; L2 = Lever 2. See the online article for the color version of this figure.

**Figure 5 fig5:**
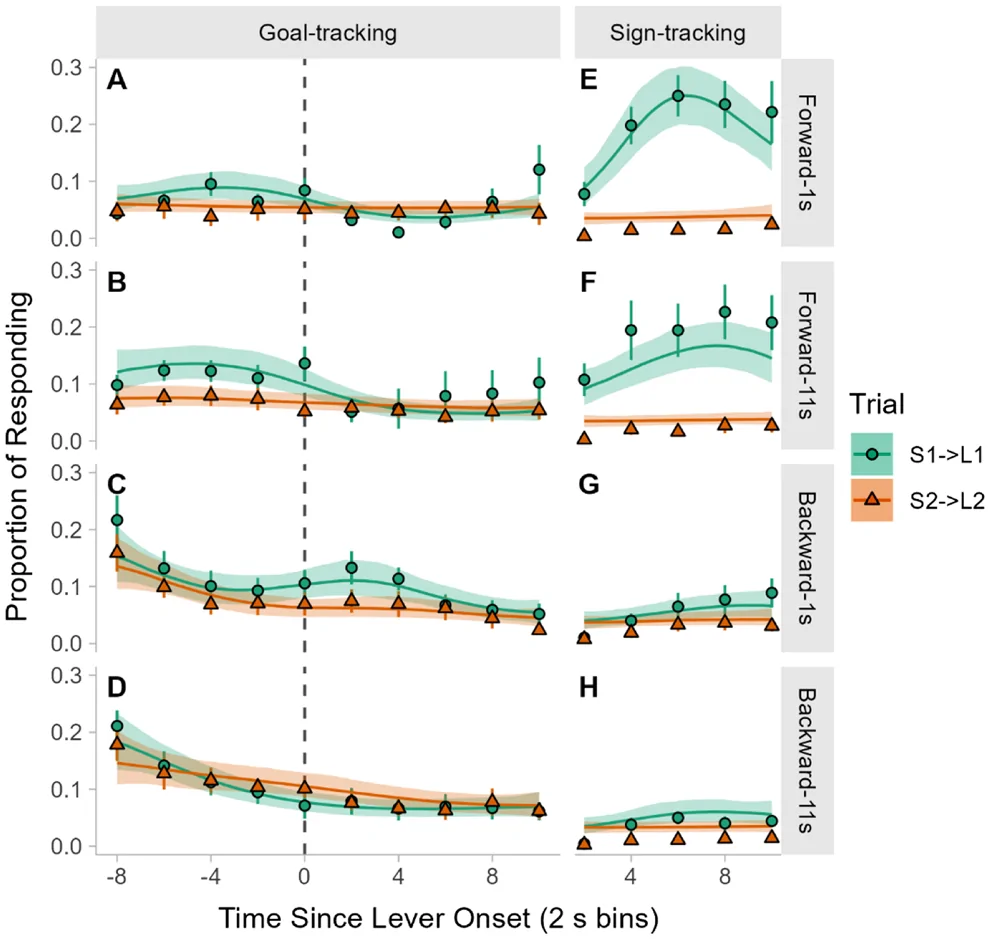
Experiment 2 *Note.* The distribution of responding across the 10-s components of the nonreinforced higher-order conditioning trials in Experiment 2 (i.e., S1→L1 and S2→L2) in four groups of rats distinguished by whether conditioning trials involved forward or backward pairings of L1 and food, and whether the interval between the L1 and food was 1 s or 11 s: forward-1s and forward-11s, backward-1s, and backward-11s. In all groups, L2 was not paired with food. Each panel shows the proportion of responding across 2-s bins, on the last six sessions of higher-order conditioning. Panels A–D show the mean proportions of goal-tracking on S1→L1 and S2→L2 trials. Panels E–H show the corresponding proportions of sign-tracking during L1 and L2. The vertical dashed lines denote the offset of the 10-s sounds (S1 and S2) and the onset of the levers (L1 and L2). Filled points denote group means and their whiskers ± the standard error of the mean. The lines represent mean posterior predictions from our statistical model, and the shaded areas surrounding them denote their 95% credible interval. S1 = Sound 1; S2 = Sound 2; L1 = Lever 1; L2 = Lever 2. See the online article for the color version of this figure.

## Footnotes

1 The mean levels of goal-tracking in group forward-11s during the late bins of L1 shown in Figure 3B do not represent accurately the performance of the group: The majority of rats in this group showed little to no goal-tracking during these later bins. Our statistical model correctly reflects this fact.

2 Navarro (2024) has developed an open-access R package (calmr) for generating simulations of HeiDI and other models of Pavlovian conditioning (https://cran.r-project.org/web/packages/calmr/index.html).
