# Erratum to “The Spatiotemporal Dynamics of Conditioned Behavior: First-Order and Higher-Order Conditioning” by Navarro et al. (2025)

**DOI:** 10.1037/xan0000411

**Published:** 2025-11-20

The article “The Spatiotemporal Dynamics of Conditioned Behavior: First-Order and Higher-Order Conditioning,” by Victor M. Navarro, Dominic M. Dwyer, and Robert C. Honey (*Journal of Experimental Psychology: Animal Learning and Cognition*, 2025, Vol. 51, No. 2, pp. 92–102, https://doi.org/10.1037/xan0000392), had the incorrect open access license listed in the author note due to a processing error. The correct open access license for the article is CC BY 4.0; https://creativecommons.org/licenses/by/4.0. The online version of this article has been corrected.

